# Sensomotoric Orthoses, Ankle–Foot Orthoses, and Children with Cerebral Palsy: The Bigger Picture

**DOI:** 10.3390/children7080082

**Published:** 2020-07-24

**Authors:** Clare MacFarlane, Robin Orr, Wayne Hing

**Affiliations:** Faculty of Health Sciences and Medicine, Bond University, Gold Coast, QLD 4226, Australia; rorr@bond.edu.au (R.O.); whing@bond.edu.au (W.H.)

**Keywords:** sensomotoric orthoses, ankle–foot orthoses, cerebral palsy, gait, gross motor skills, quality of life, children

## Abstract

Ankle–foot orthoses (AFOs) and sensomotoric orthoses (SMotOs) are two—clinically relevant, yet under researched—types of lower limb orthoses used in children with cerebral palsy (CP). Quality of life is a state of complete physical, mental and social well-being and not merely the absence of disease or infirmity. Evaluating the effect of these two types of orthoses on quality of life in children with CP has not been reported on. The aim of this case study series was to synthesise and enrich the volume of evidence reported to inform real world applications of SMotO use in children with CP. Participants recruited were children with CP who performed the Berg Balance Scale, Timed Up-and-Go, the Gross Motor Function Measure and/or the Edinburgh Visual Gait Score in AFOs, SMotOs and barefoot where able. Qualitative data included videos of gait, a questionnaire and pedographs. Eight participants completed 39 quantitative and six qualitative measures, with the Edinburgh Visual Gait Score (EVGS) reporting the highest response. A general improvement was seen in gross motor skills and gait when wearing the SMotOs compared to AFOs and some parents reported that SMotOs were preferred. The reader is able to correlate the quantitative results with the qualitative evidence presented.

## 1. Introduction

Ankle–foot orthoses (AFOs) and sensomotoric orthoses (SMotOs) are two types of lower limb orthoses. The benefits of using AFOs in children with cerebral palsy (CP) has been well documented over the years. AFOs are designed to affect body structure [1], support normal joint alignment and mechanics, provide variable range of motion (ROM) when appropriate, facilitate function [1,2,3,4], stabilise the ankle/foot complex [5] and enable a continuous Achilles/gastrocnemius stretch [6,7,8]. Along with joint alignment, other improvements that may be seen through the use of AFOs are improvements in walking efficiency [9,10], the position of the foot for function [11], and improvements in gait function and pain prevention [12]. Common types of AFOs seen in the literature are solid AFO (SAFO), hinged AFO (HAFO), and dynamic AFOs [5,13,14].

SMotO is a clinically relevant, yet under researched, orthoses option used in the same population. Unlike AFOs, SMotOs provide a different approach to the management of gait in children with CP. Wegner et al. [15], describe one adaptation theory as ‘elements’ on the foot orthoses (e.g., forefoot valgus posting or lateral rearfoot padding) increasing local pressures, which are detected by cutaneous receptors, muscle spindles or Golgi apparatus on musculotendinous structures in the foot of the tibialis posterior, peroneus brevis and the lumbricals/quadratus plantae. Depending on these individual pressure bumps’ height and placement, the muscles can be activated or restricted [16,17]. CP affects the different areas of the brain, thereby interrupting signals sent to the muscles. The SMotOs work via the idea that the signals are being sent from the muscles back up to the spinal cord through activation of the Golgi bodies, therefore signalling muscles to respond to stimuli [15].

Quality of life (QoL) is defined by the World Health Organisation [18]: “health is a state of complete physical, mental and social well-being and not merely the absence of disease or infirmity. The enjoyment of the highest attainable standard of health is one of the fundamental rights of every human being without distinction of race, religion, political belief, economic or social condition”. With regard to CP, factors relating to QoL can include the child (age, gender, and severity of the disease; comorbidity and complications), family (socioeconomic status, relationships and support, coping mechanisms, parenting style, and knowledge about the disease) and the availability of management and rehabilitation services, as well as other environmental factors [19]. In a QoL study by Dickinson et al. [20], children with CP were investigated using KIDSCREEN (an instrument with scores in 10 domains) [21] and, through a comparison of the least and most able groups, severely limited self-mobility was significantly associated with a reduced mean score for physical wellbeing (7.6, 95% CI 2.7–12.4, *p* = 0.002), and pain was common and associated with a lower QoL in all domains. They concluded that physical impairments and presence of pain were responsible for variations (3% and 7%, respectively) in QoL. Therefore, a child’s pain should be carefully assessed. When physical impairment impacts function, thus affecting QoL, therapists would likely make improving function a goal area for therapy. Independent walking is a typical goal of rehabilitation in children with CP, but this expectation can lead to frustration in parents and children, many of whom feel that they are more mobile and more functional when using assistive devices [22] versus completely independent. 

Evaluating the effect of these two types of orthoses on quality of life in children with CP has not been reported on. Creating a ‘real life’ picture of particular ‘cases’ or participants in a mixed method case series study can bring depth to understanding both the clinical relevance and impact of an intervention on certain aspects of life. Although case series represent a low level of evidence (IV) and have methodological limitations with regard to making causal inferences about the relation between treatment and outcome [23], Murad et al. [24] suggested that when no other higher level of evidence is available, decision making can be informed using evidence derived from case reports and case series. 

There is one published paper into the effect of SMotO on gait [25], and none on the effects of SMotO on gross motor skills and quality of life in children with CP. To provide a more complete picture of these complex children, a need to merge these studies in a select group of participants was found. 

Therefore, noting the lack of literature in this field, the aim of this case study series was to synthesise and enrich the volume of evidence reported to inform real-world applications of SMotO use in children with CP. This case series also aims to demonstrate the impact of SMotOs and AFOs on function, movement and quality of life in the individual, in a way that is clinically relatable. 

## 2. Materials and Methods

### 2.1. Study Design

Ethical process: Ethical approval was obtained through the Bond University Research and Ethics Committee (Approval RO-1835). Consent was gained from Clinic Directors in both private practice settings. Parents/caregivers were given an explanatory statement and consent form, both of which were read and completed before data collection took place. Consent was gained for video and image capture.

This study was a retrospective mixed method design, with a combination of both quantitative and qualitative outcome measures collected. Outcome measures were undertaken in two separate clinic locations, as well as six home settings due to the families being unable to travel. These settings were selected as they were familiar to the child and allowed the parents and/or siblings to be present throughout the testing. Relevant, pertinent participant qualitative information from the questionnaire (Q’AIRE) was extracted and combined with the correlating participants quantitative measurements to create the case series and is described in greater detail below. 

### 2.2. Participants 

Recruitment and inclusion criteria: Participants were children with CP recruited by convenience sampling through two private therapy practices (Therapies for Kids and NAPA Centre, Sydney, NSW, Australia). The inclusion criteria were: (a) diagnosis of CP with any Gross Motor Function Classification System (GMFCS) level, (b) using SMotOs/AFOs (or have used them) and completed the wearing in process, and (c) no surgery in past six weeks.

### 2.3. Intervention

All participants brought their own SMotOs and AFOs. The AFOs were all made from polypropylene with Velcro straps holding the foot in place. The SMotOs were custom made for each child from ethyl vinyl acetate (EVA). Each participant used SMotOs and/or AFOs whilst participating in outcome measures.

### 2.4. Quantitative Outcome Measures 

The quantitative section of the case series process included the principal researcher assessing each participant as able using Timed Up-and-Go (TUG), the Berg Balance Scale (BBS), the Gross Motor Function Measure (GMFM-88), and/or the Edinburgh Visual Gait Score (EVGS). Each outcome measure was performed as the child was able, in any order deemed appropriate, and in any order of orthoses. For example, one child came in wearing AFOs and wanted to walk around the clinic; therefore, the EVGS in AFOs was assessed first. This child then became interested in some static activities; therefore, the GMFM in AFOs was performed next. Data collection continued for as many outcome measures as was possible for each of the participants’ and within their ability and tolerance levels.

### 2.5. Qualitative Outcome Measures

Three styles of qualitative evidence were included: written feedback from parents compilated from the Q’AIRE, images of pedographs (pre- and post-SMotO) and/or video images of gait.

The Standards for Reporting Qualitative Research were followed [26]. A qualitative phenomenological approach was employed through a questionnaire-based survey. The Q’AIRE was designed to establish the effect of lower limb orthoses in current day to day QoL. This Q’AIRE was also undertaken to determine how the SMotO and AFO affect the child’s function, as reported by parents. The Q’AIRE was emailed to multiple families after participating in quantitative data collection. Qualitative video images of a typical gait pattern were taken with the participant barefoot (where able), in AFOs and SMotOs. Video images were taken with a handheld device (Apple iPhone 7s, Apple Inc., Cupertino, CA, USA). The videos were taken in whichever location the outcome measures were recorded—either in clinic or at the participant’s home—while the participant mobilised at a self-directed pace, using their usual prescribed walking aid (where necessary). Pedographs were supplied by the pedorthist. 

## 3. Results

### 3.1. Participants and Outcome Measures

Data for eight participants (male: *n* = 7: female: *n* = 1) were collected. Participant 2 had EVA heel wedges on their SAFO to encourage weight through the heel, mimicking heel strike. The eight participants demonstrated a large range of physical abilities with reported GMFCS levels of I (*n* = 1 participant), II (*n* = 2 participants), III (*n* = 2 participants) and IV (*n* = 3 participants). The age range was three to 13 years (average age = 7 ± 3.7 years). Overall, there were 39 quantitative and six qualitative measures collected (Table 1). The EVGS demonstrated the highest response. Please note that, in videos, participants were previously coded (embedded in video) and, as such, may display a different participant number to the current number. The podiatrist and pedorthist who prescribed and fabricated the SMotOs provided pedograph images of two participants’ footprints (7 and 8) before and after the use of SMotOs.

### 3.2. Case Series

Data for each of the eight retained participants is presented below as individual cases.

**Figure 1:**
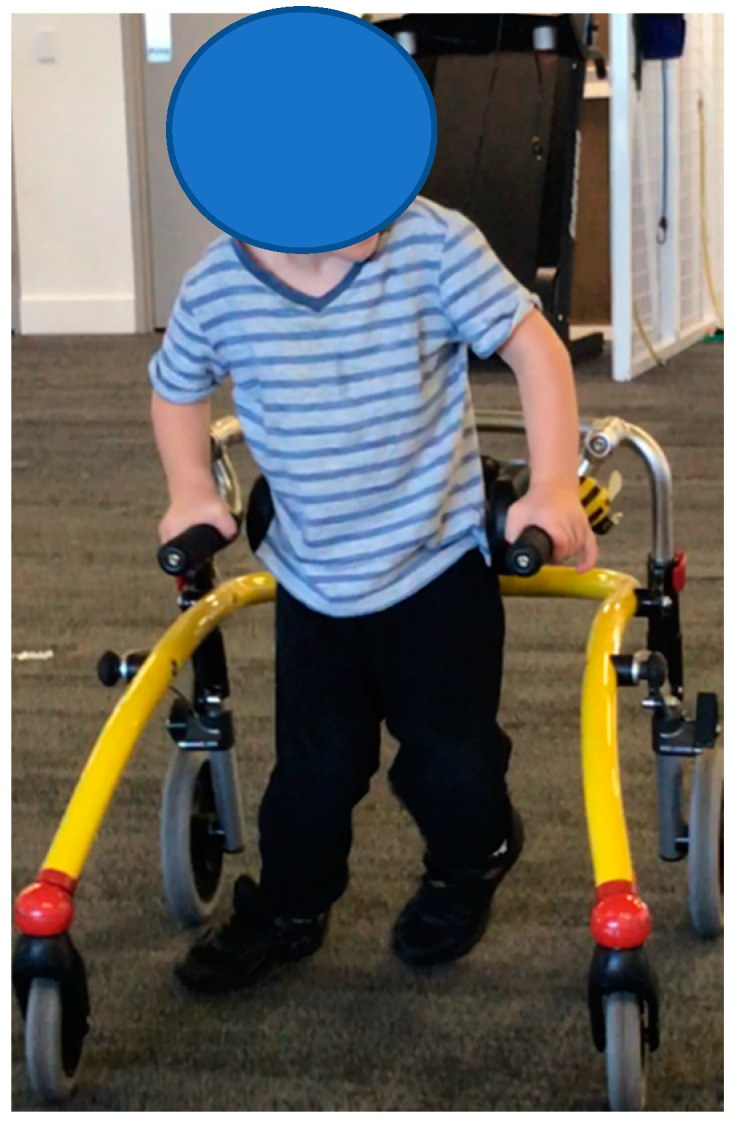
Participant 1.

**Participant 1**: Four-year-old male child with spastic diplegic CP, GMFCS III. The participant mobilises with a reverse walker. Participant 1 (Figure 1) demonstrated better scores in TUG, GMFM-88 and EVGS when in SMotO, likely due to the dynamic nature of the SMotOs being used in dynamic outcome measures (Table 2). Participant 1 displayed a better score in the BBS when in AFOs, likely due to the bracing effect of AFOs. 

As per response from the Q’AIRE, the participant’s mother reported that “I have been advised by some of our health care professionals that (my) son’s gait is better in his AFOs than in Piedro (supportive disability shoe) with SMotO”. This statement is contradicted by the EVGS results (Table 2). The mother of participant 1 did not give consent for video images of his gait. 

**Figure 2:**
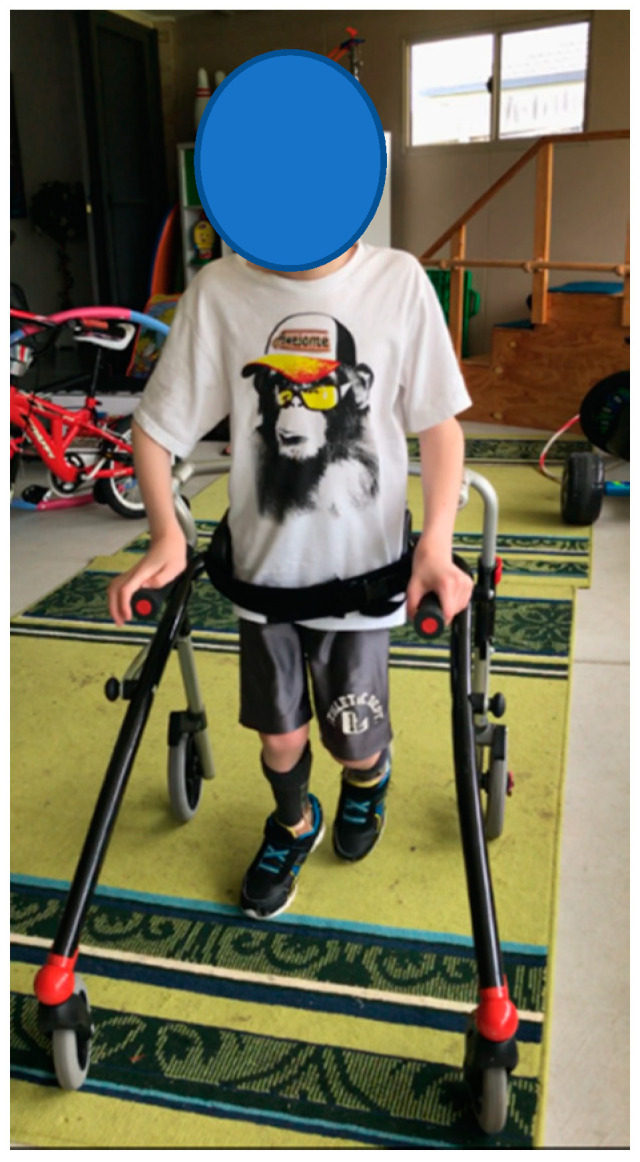
Participant 2.

**Participant 2:** Eight-year-old male child with spastic quadriplegic CP, GMFCS III. The participant mobilises with a reverse walker. Participant 2 (Figure 2) performed better in the TUG, GMFM-88 and EVGS when wearing SMotOs, likely due to the dynamic nature of the SMotOs being used in dynamic outcome measures (Table 3). Interestingly, the BBS reported the same score for both orthoses. Correlating videos (in DropBox folder link below) highlighting the participant’s gait in SMotO, AFO and barefoot (as labelled) for ‘Participant 2’ have been provided for reference. 

The participant’s mother reported, as per the Q’AIRE, that “the SMotOs have been great for the stepping, sit to stand, pull to stand. Anything where he gets to feel the ground with the ankle movement has been the biggest bonus. Once I get some more supportive shoes to go with these then this will be the best. His Piedros still weren’t helpful but we are looking at custom made ones to help this”. 

https://www.dropbox.com/sh/tfcrp9c1dxwmbn0/AAB9FSgGPunYpi8uCDDxZKpAa?dl=0

**Figure 3:**
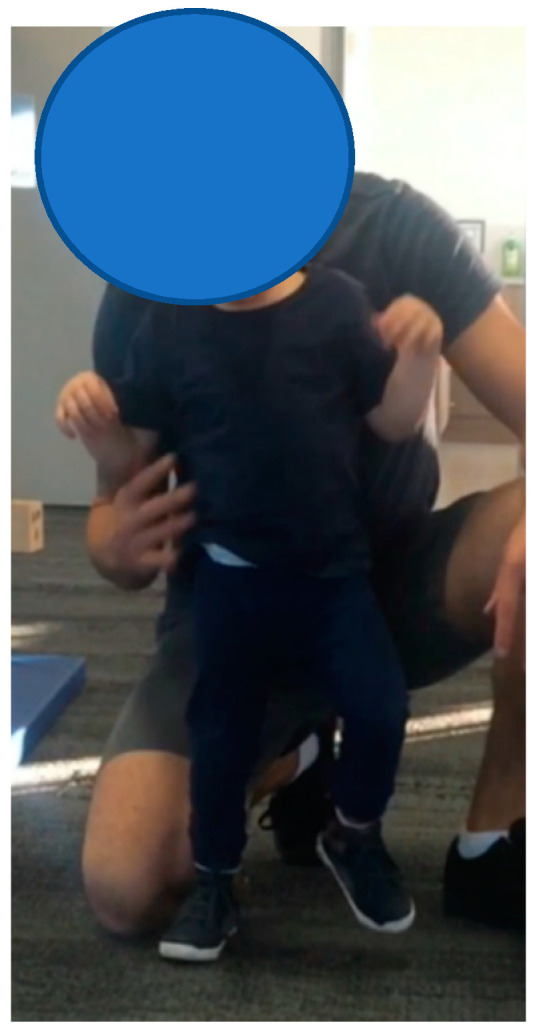
Participant 3.

**Participant 3:** Four-year-old boy with spastic diplegic CP, GMFCS II. Participant 3 (Figure 3) demonstrated improved scores in the BBS, GMFM-88 and EVGS when wearing SMotOs compared to AFOs (Table 4). The GMFM-88 demonstrates a change of 6%, which is reported as a clinically important change in score. Correlating videos (in DropBox folder link below) highlighting the participant’s gait in SMotO, AFO and barefoot (as labelled) for ‘Participant 3’ have been provided for reference. 

The mother of Participant 3 reported, as per the Q’AIRE, that her “son is much more comfortable in SMotOs and finds it easier to manoeuvre his body and is much more willing to get up and try new things with them on because they’re not as bulky”.

https://www.dropbox.com/sh/ew4mbh9elsgpk63/AACXVALqehvueHW0bVabgQHaa?dl=0

**Figure 4:**
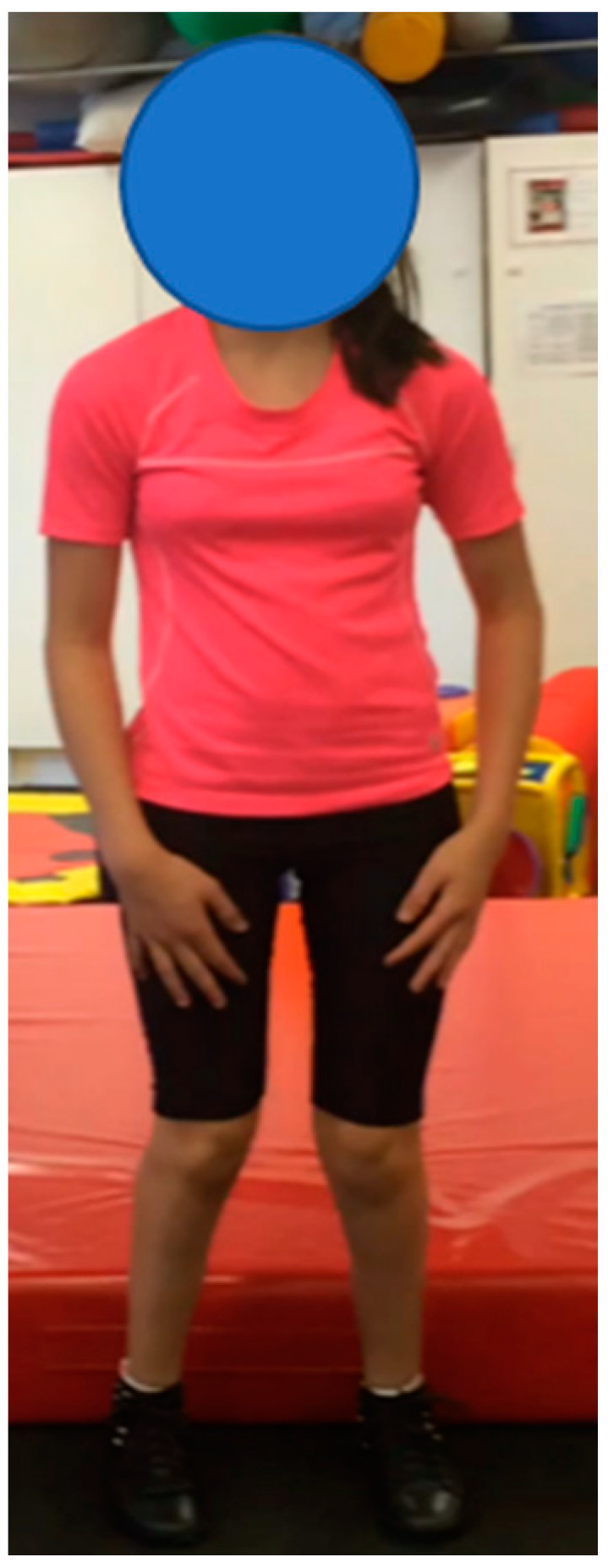
Participant 4.

**Participant 4:** Thirteen-year-old girl with spastic quadriplegic CP, GMFCS II. Participant 4 (Figure 4) visually appeared to walk well in AFOs, but the results of the EVGS (Table 5) demonstrated a notable difference in the quality of her gait pattern when wearing AFOs compared to SMotO. Her GMFM-88 total score did not display a large difference in scores between orthoses, indicating that neither orthosis demonstrates an increased effect on gross motor skills compared to the other. Correlating videos (in DropBox folder link below) highlighting the participant’s gait in SMotO, AFO and barefoot (as labelled) for ‘Participant 4’ have been provided for reference. Participant 4 did not complete the Q’AIRE. 

https://www.dropbox.com/sh/1gd73f6b3uufwzh/AAAQM-uMtXsmpPieC86bStp9a?dl=0

**Figure 5:**
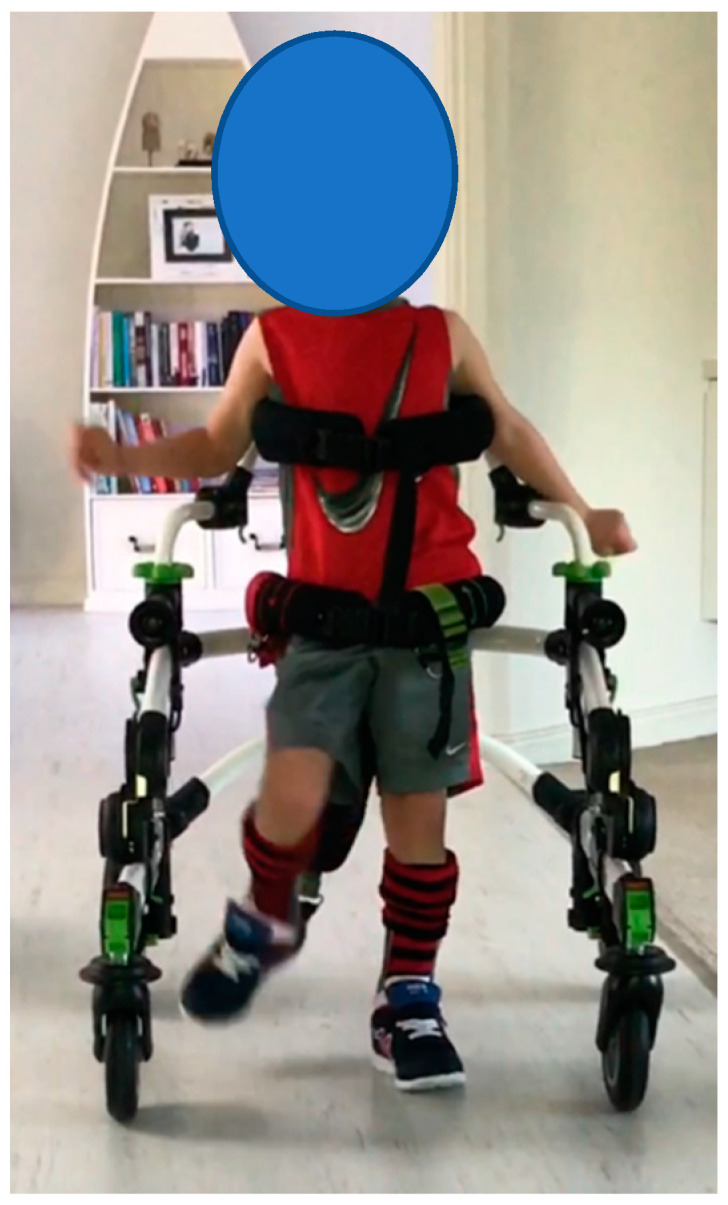
Participant 5.

**Participant 5:** Four-year-old boy with dystonic quadriplegic CP, GMFCS IV. The participant mobilises with a supportive reverse walker. Participant 5 (Figure 5) was physically affected by his dystonia and used a gait trainer to mobilise. He was unable to participate in any other outcome measures. Despite this limitation, the video images and EVGS both demonstrated the clear differences in his gait between barefoot, AFO and SMotOs. The qualitative evidence highlighting the participant’s gait in the three conditions (correlating videos in DropBox folder link below) is supported by the results from the EVGS (Table 6). 

https://www.dropbox.com/sh/8uolo7lhk5v5g2e/AAAV_tK_KujMt0Sew6t__p5ba?dl=0

**Figure 6:**
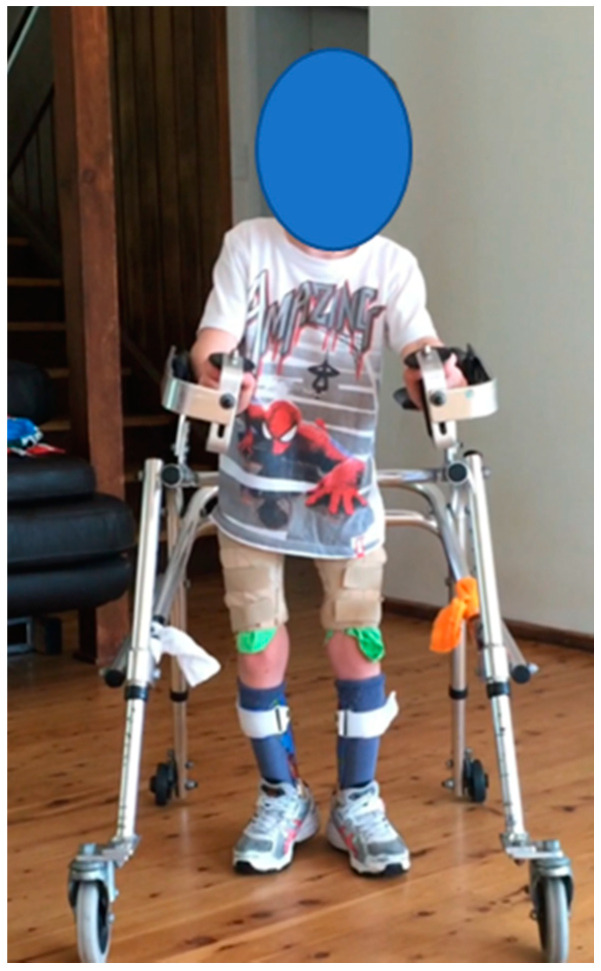
Participant 6.

**Participant 6**: Five-year-old boy with spastic quadriplegic CP, GMFCS IV. The participant mobilises with a reverse walker and hip abduction brace. Participant 6 (Figure 6) struggled to walk without the support of his orthoses, walking frame and abduction brace. The results from the GMFM-88 (Table 7) showed a mild difference in scores between orthoses. Both the quantitative measure (EVGS) and qualitative images (video as per link below) demonstrated a difference in the quality of movement between orthoses and barefoot. 

Correlating videos (in DropBox folder link below) highlighting the participant’s gait in SMotO, AFO and barefoot (as labelled) for ‘Participant 6’ have been provided for reference. Participant 6 did not complete the Q’AIRE.

https://www.dropbox.com/sh/khmll6jwoumlpnv/AAAAgXhzhgX4PJGFpjRX6FR6a?dl=0

**Figure 7:**
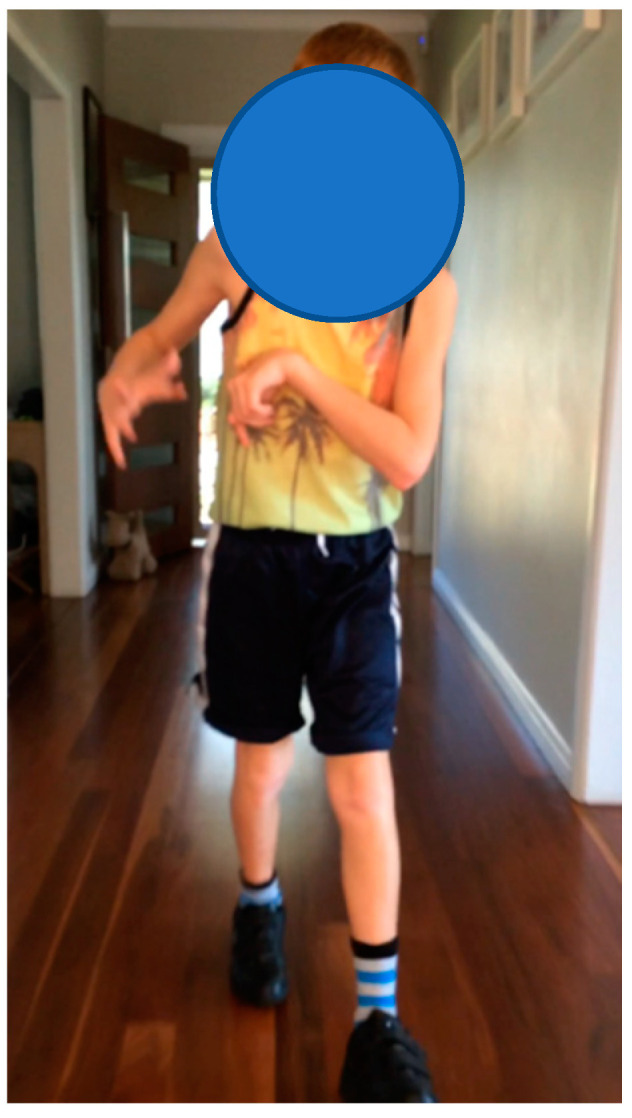
Participant 7.

**Figure 8:**
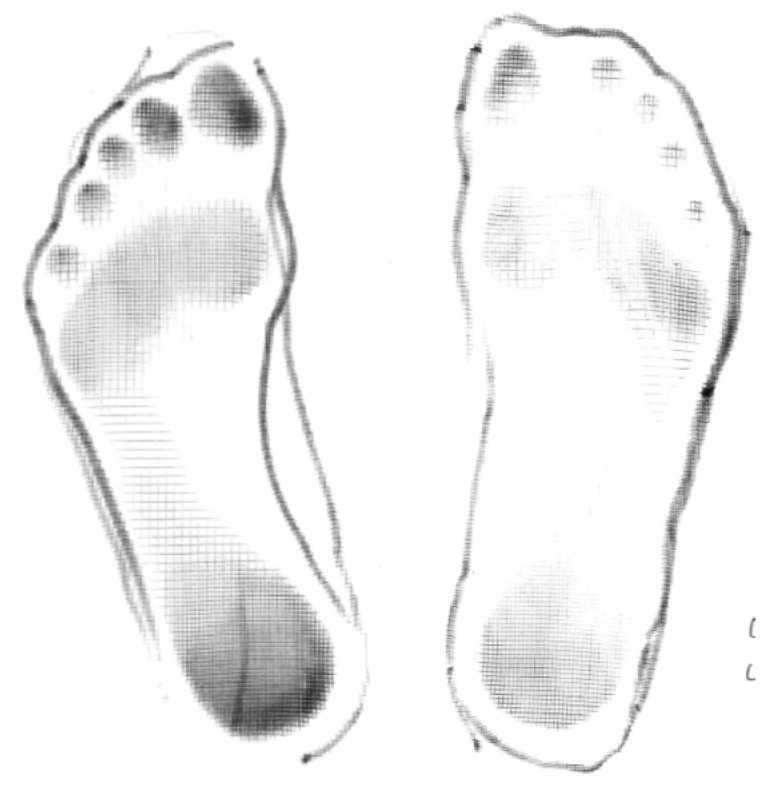
Participant 7 left and right initial pedograph footprint (2016).

**Figure 9:**
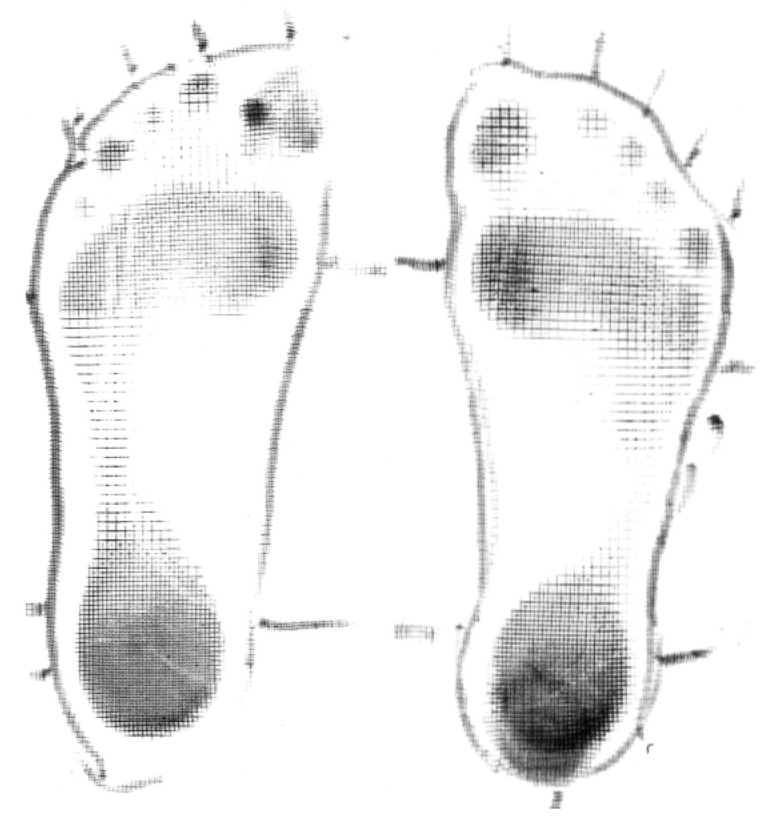
Participant 7 left and right final pedograph footprint (2017).

**Participant 7:** Twelve-year-old boy with spastic dystonic quadriplegic CP, GMFCS I. Participant 7 (Figure 7) was independently mobile with and without shoes. Participant 7 demonstrated improved alignment and stability when he wore SMotO as per EVGS score (Table 8) compared to barefoot. The images from the pedographs (Figure 8 and Figure 9) demonstrated weightbearing changes pre-SMotO and one year after using SMotO, especially through the right foot.

Correlating videos (in DropBox folder link below) highlighting the participant’s gait in SMotO and barefoot (as labelled) for ‘Participant 7’ have been provided for reference. Participant 7 did not complete any other outcome measures.

https://www.dropbox.com/sh/i25vyb9jnd5v6f2/AABj5pBSJ4MNBj9kk4LPDbRSa?dl=0

**Figure 10:**
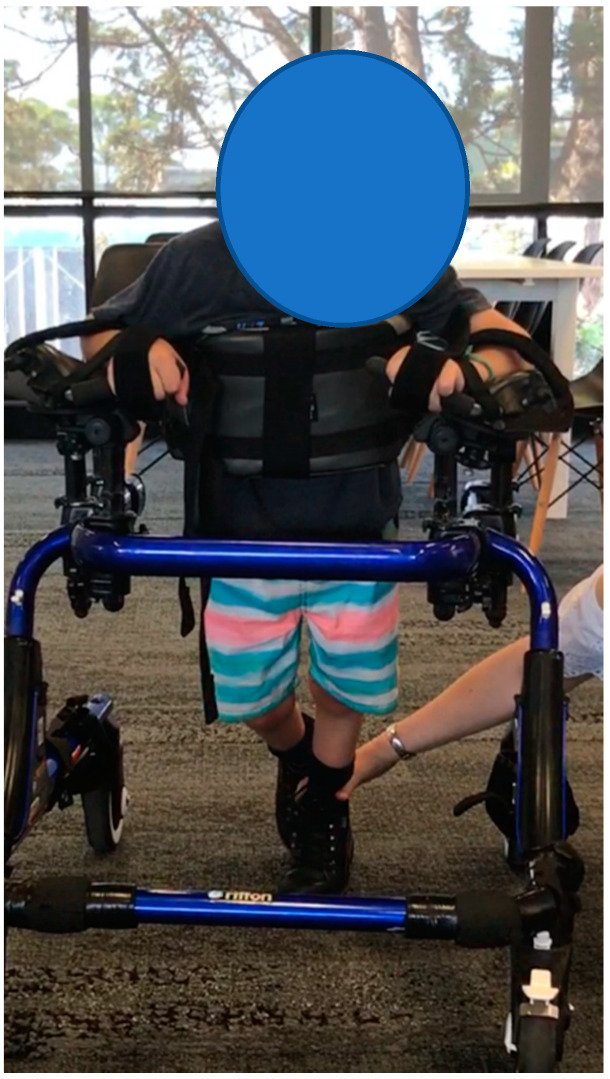
Participant 8.

**Participant 8**: Six-year-old boy with dystonic quadriplegic CP, GMFCS IV. The participant mobilises with assistance in a supported walker. Participant 8 (Figure 10) was originally prescribed SAFOs then HAFOs despite not having any restriction in his ankle range of motion. He was able to bear weight with support and walks in a walker. He uses SMotOs in a Piedro shoe. Participant 8 was unable to complete any of the quantitative outcome measures due to his severe dystonia. From the Q’AIRE, mother reported “for children with CP—it appears there is a standard practice/framework for which children are expected to have/need. AFOs are one of these. I had to suggest my child transition from SAFO to HAFO. It was not suggested to us. They provide better support and ankle flexibility”. 

**Figure 11:**
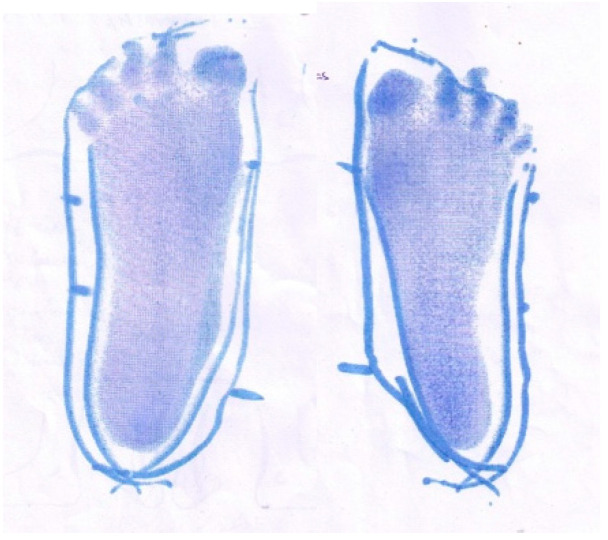
Participant 8 left and right initial pedograph footprint (15 June 2016).

**Figure 12:**
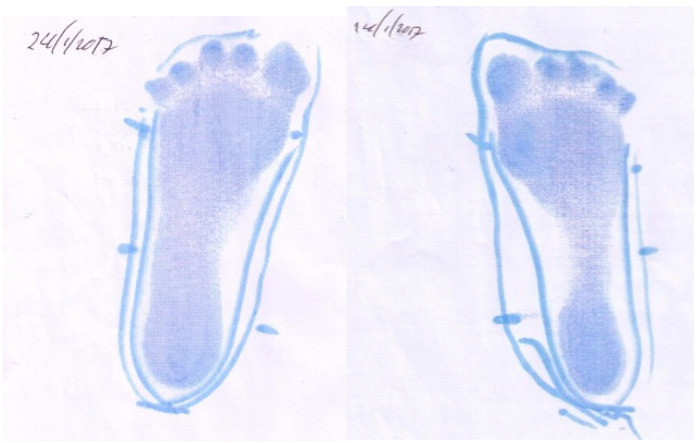
Participant 8 left and right final pedograph footprint (24 January 2017).

Figure 11 and Figure 12 demonstrate the changes seen (over a seven-month period) in the muscle activation of his foot when wearing the SMotOs. These pedograph images corroborate the theory of the muscles learning to activate and support the foot, despite his CP diagnosis. 

## 4. Discussion

Collecting a range of data in this population is challenging due to participants’ age, level of disability, cognitive comprehension, inability to process instructions or give feedback, poor motor planning, and general behaviour. As such, data can often be incomplete when multiple outcome measures are collected. Given the challenges of collection and the volume and variety of data collected in this program of research, the use of a mixed method approach allowed for the collation of both quantitative and qualitive data to enrich the research findings. Furthermore, the inclusion of qualitative information to create a more holistic viewpoint of the intervention findings allowed the caregivers to validate and express their experiences.

The individual outcome measure results suggested a difference between areas of static and dynamic movement with SMotO and AFO. The general observed trend was increased static balance whilst wearing the AFO, and general improved ability (score) with dynamic movement when wearing the SMotO. This result is encouraging as a foundation to warrant further investigation into the use of SMotOs in this population for gross motor skills.

It appears, through both the qualitative and quantitative results, that children with CP have some preference for using SMotOs. In support of this, when looking at the Q’AIRE qualitative data, parents tended towards more positive comments regarding the use of SMotOs when compared to AFOs for gross motor skills and ease of use. In addition, it was identified that families do not have follow up appointments to reassess gait with AFOs and the impact of the AFO on gross motor skills. Clinically, it may also be beneficial to implement a follow-up timeline to reassess the effect of orthoses prescribed.

Using a clinic where a large number of clients reside interstate or internationally leaves the study somewhat lacking a significant number of potential participants, hence the low numbers of participants and inconsistent numbers of completed outcome measures. Some families had agreed to participate in all areas of the study but were unable to commit to the time required to complete the assessment, unable to participate due to distance or were unable to complete assessment due to the child’s behaviour. A limiting factor of participants completing all outcome measures was the inability of the child to comprehend complex instructions relating to outcome measures, and participation was also affected due to participants’ inability to process verbal instructions. However, the use of a gait aid did allow participants with lower functioning GMFCS levels to participate in the EVGS with individual results compared between orthoses. Another limitation is the bias towards the male gender, but future research could include a more even split between the genders.

### Future Research Directions

While we acknowledge that this small case series demonstrates a minute part of the population, it does provide some important insights into the child as a ‘whole picture’ versus statistically based evidence alone. Future research could include creating a more specific assessment to determine the ability of the child, the family goals, and to investigate these in relation to the ability of SMotOs and AFOs to meet these goals, in order to create a more individualised, child-centred, goal-driven approach to orthoses prescription.

## 5. Conclusions

The aim of the current case series was to synthesise different participants’ qualitative and quantitative evidence to support the volume of evidence in this thesis. It was also aimed at informing real-world applications of SMotO in children with CP using clinically relevant outcome measures relating to gait and gross motor skills orthoses prescription and what the best course of action for families and health professionals is.

It was concluded, based on the overall results of this case series, that a general improvement was seen in gross motor skills and gait when wearing the SMotOs compared to AFOs and they were preferred by some parents. By displaying video images of participants’ gait in AFO, SMotO and barefoot, viewers are able to correlate quantitative results with the qualitative evidence of performance.

## Figures and Tables

**Table 1:** Participant quantitative and qualitative outcome measure responses.

Outcome Measure		Intervention		
Quantitative		SMotO	AFO	Barefoot
	EVGS	7	6	2
	GMFM-88	5	5	0
	BBS	4	4	0
	TUG	3	3	0
Qualitative	Responses			
	Q’AIRE	4		
	Pedograph	2		
	Videography of gait	6		

SMotO: sensomotoric orthoses; AFO: ankle-foot orthoses; EVGS: Edinburgh Visual Gait Score; BBS: Berg Balance Scale; TUG: Timed Up-and-Go; Q’AIRE: Questionnaire.

**Table 2:** Participant 1 outcome measure comparative results between and sensomotoric orthoses (SMotOs) and ankle–foot orthoses (AFOs).

Outcome Measure	SMotO	AFO
TUG (s)	13.8 s	17 s
BBS (/56)	15	12
GMFM-88 (%)	73.51	71.17
EVGS	25 (total L & R)	38 (total L & R)

**Table 3:** Participant 2 outcome measure comparative results between SMotO and AFO.

Outcome Measure	SMotO	AFO
TUG (s)	41.13	44.37
BBS (/56)	7	7
GMFM-88 (%)	69.11	64.41
EVGS	30 (total L & R)	41 (total L & R)

**Table 4:** Participant 3 outcome measure comparative results between SMotO and AFO.

Outcome Measure	SMotO	AFO
TUG (s)	Unable to follow direction	
BBS (/56)	17	13
GMFM-88 (%)	85.51	79.51
EVGS	8 (total L & R)	15 (total L & R)

**Table 5:** Participant 4 outcome measure comparative results between SMotO and AFO.

Outcome Measure	SMotO	AFO
TUG	11.33	10.13
BBS	39.00	37.00
GMFM-88 (%)	91.29	92.00
EVGS	15 (total L & R)	31 (total L & R)

**Table 6:** Participant 5 comparative Edinburgh Visual Gait Score (EVGS) results between barefoot, AFO and SMotO.

Outcome Measure	Barefoot	AFO	SMotO
EVGS	51 (total L & R)	30 (total L & R)	17 (total L & R)

**Table 7:** Participant 6 outcome measures comparative results between SMotO and AFO.

Outcome Measure	SMotO	AFO
GMFM-88 (%)	49.52	47.67
EVGS	9 (total L & R)	25 (total L & R)

**Table 8:** Participant 7 EVGS results SMotO and barefoot.

Outcome Measure	SMotO	Barefoot
EVGS	3 (total L & R)	13 (total L & R)
